# A Novel Framework for the Optimization of Simultaneous ThermoBrachyTherapy

**DOI:** 10.3390/cancers14061425

**Published:** 2022-03-10

**Authors:** Ioannis Androulakis, Rob M. C. Mestrom, Miranda E. M. C. Christianen, Inger-Karine K. Kolkman-Deurloo, Gerard C. van Rhoon

**Affiliations:** 1 Department of Radiotherapy, Erasmus MC Cancer Institute, University Medical Center Rotterdam, 3015 Rotterdam, The Netherlands; m.christianen@erasmusmc.nl (M.E.M.C.C.); i.kolkman-deurloo@erasmusmc.nl (I.-K.K.K.-D.); 2 Department of Electrical Engineering, Eindhoven University of Technology, 5600 Eindhoven, The Netherlands; r.m.c.mestrom@tue.nl; 3 Department of Radiation Science and Technology, Delft University of Technology, 2629 Delft, The Netherlands

**Keywords:** hyperthermia, induced, brachytherapy, prostatic neoplasms, interstitial hyperthermia, treatment plan optimization, prostate, thermoradiotherapy, linear quadratic model, biological modeling

## Abstract

**Simple Summary:**

ThermoBrachyTherapy, a combination therapy where radiation and heat are simultaneously applied using needle-shaped applicators from within the target, is a potentially very effective treatment for prostate cancer. When radiation and thermal therapies are applied, the dose coverage of each treatment is preplanned without considering the combined effect of the two dose distributions. In this study, we propose a method to automatically plan the thermal dose in such a treatment, based on the combined effect with the radiation. Furthermore, we apply the method on 10 patients and compare the treatment to a brachytherapy-only treatment plan. In this way, we show that, with properly optimized ThermoBrachyTherapy, we can provide equivalent combined dose coverages to the prostate, while reducing the dose delivered to critical organs surrounding the prostate, which might translate to reduced toxicity of the treatment.

**Abstract:**

In high-dose-rate brachytherapy (HDR-BT) for prostate cancer treatment, interstitial hyperthermia (IHT) is applied to sensitize the tumor to the radiation (RT) dose, aiming at a more efficient treatment. Simultaneous application of HDR-BT and IHT is anticipated to provide maximum radiosensitization of the tumor. With this rationale, the ThermoBrachyTherapy applicators have been designed and developed, enabling simultaneous irradiation and heating. In this research, we present a method to optimize the three-dimensional temperature distribution for simultaneous HDR-BT and IHT based on the resulting equivalent physical dose (*EQD_phys_*) of the combined treatment. First, the temperature resulting from each electrode is precomputed. Then, for a given set of electrode settings and a precomputed radiation dose, the *EQD_phys_* is calculated based on the temperature-dependent linear-quadratic model. Finally, the optimum set of electrode settings is found through an optimization algorithm. The method is applied on implant geometries and anatomical data of 10 previously irradiated patients, using reported thermoradiobiological parameters and physical doses. We found that an equal equivalent dose coverage of the target can be achieved with a physical RT dose reduction of 20% together with a significantly lower *EQD_phys_* to the organs at risk (*p*-value < 0.001), even in the least favorable scenarios. As a result, simultaneous ThermoBrachyTherapy could lead to a relevant therapeutic benefit for patients with prostate cancer.

## 1. Introduction

High-dose-rate brachytherapy (HDR-BT) is a well-established treatment option in localized prostate cancer treatment [1]. Radiobiological clinical data have shown that prostate cancer, in contrast to most tumor sites, has a very low *α*/*β* ratio (*α*/*β* = 0.9–2.2 Gy) [2]. This is a value very close to or lower than the *α*/*β* of nearby organs at risk (OAR), with the urethra estimated at an *α*/*β* = 0.5–1 Gy [3] and the rectum estimated at an *α*/*β* = 1.6–3.1 Gy [4], depending on the considered toxicity endpoint. This very low α/β ratio is the reason that radiotherapy for prostate cancer is aimed towards hypofractionation, with extensive use of HDR-BT as a boost to external beam radiation therapy (EBRT) or even as a standalone therapy (monotherapy) [5,6].

In HDR-BT monotherapy for prostate cancer, several clinical trials have shown that even ultrahypofractionated treatments of 2–4 fractions lead to excellent overall disease-free survival in low-risk and favorable intermediate-risk cancer patients [6,7,8]. On the other hand, the treatments are still linked with genitourinary and gastrointestinal toxicities [9]. Moreover, further reduction to a single fraction treatment has shown very poor results in multiple studies, with the reasons for those poor results not yet clear. Furthermore, ultrahypofractionated HDR-BT monotherapy is currently not recommended in higher-risk patients [10,11].

The abovementioned drawbacks could be overcome if the treatment could further escalate the radiation dose in the prostate without affecting the OAR, or if the same dose could be reached in the prostate with a reduced dose in the OAR. A way to achieve this could be by selective target radiosensitization. One of the most potent sensitizers to radiation is hyperthermia [12,13]. It has also been shown in clinical data that hyperthermia reduces the α/β of tumors [14] and, hence, makes the tumors more favorable to hypofractionation. This is also evident in multiple in vitro experiments on specific prostate cancer cell lines [15,16,17]. Other than that, the ability of hyperthermia to increase perfusion, increase reoxygenation, and overcome radiation-resistant hypoxia [18] could enable a reinvestigation of single fraction treatments, since the lack of reoxygenation and hypoxic cells are presumed to be a possible cause of failure, according to Morton and Hoskin [19].

Together with HDR-BT, interstitial hyperthermia (IHT) can be used to sensitize the target [20]. This is especially convenient if the same catheters used for the HDR-BT treatment can also be used for the IHT application. IHT has been applied in various early clinical trials [21,22] and, lately, in a phase II clinical trial ongoing for salvage prostate cancer treatment [23], where three fractions of IHT (1 h at 40–43 °C) followed by 10 Gy HDR-BT were applied over three weeks.

Historical biological research has clearly shown that thermal radiosensitization depends on the time interval between radiation and hyperthermia, with the highest radiosensitization reached at simultaneous (i.e., radiation during hyperthermia) application of the two modalities [13,24]. Based on this rationale, we have developed novel ThermoBrachytherapy (TBT) applicators that enable the simultaneous application of HDR-BT and capacitive coupled radiofrequency (CC-RF) interstitial heating [25]. The improved temperature-related technical characteristics of these applicators are described in our earlier publication [25].

The challenge in simultaneous thermal radiosensitization is that, according to Overgaard [26], normal tissue might be sensitized as much as the target tissue. Hence, in order to reach a therapeutic benefit with simultaneous application of the two modalities, both treatment modalities need to be confined to the target as much as possible [27]. In prostate cancer treatment, this is a challenging task, with OAR very close to (rectum, bladder) or in direct contact with (urethra) the target volume. Therefore, very precise treatment plan optimization is needed to reach a therapeutic benefit, taking into account the combined effect of both hyperthermia and radiation.

In recent years, substantial progress has been made in theoretical modeling to calculate and quantify the combined effect of radiation and hyperthermia [28,29,30,31]. Most notably, this has resulted in the temperature-dependent linear-quadratic (TDLQ) model [29] and its extended version, including direct temperature-induced cytotoxicity [30]. While these models have been used to evaluate existing treatment plans retrospectively, there has been no attempt for pretreatment plan optimization based on the combined effect of radiation and hyperthermia.

In general, research on IHT treatment plan (IHT-TP) optimization is limited and rarely applied in clinical practice [20]. In [32], Chen et al. proposed an automated optimization algorithm for ultrasound-based IHT-TP. In [33], Salgaonkar et al. validated temperature superpositioning for faster optimization of ultrasound-based IHT-TP. In [34], Kok et al. proposed a framework for fast automated temperature optimization using basic temperature-based objective functions that can also be applied on CC-RF IHT. In [35], we validated a highly accurate fast calculation method for the power deposition of the TBT applicators. The next step in producing fast IHT-TP is to automate the treatment planning process.

This study presents the framework to optimize the IHT-TP parameters based on the equivalent RT dose resulting from the TDLQ model. This optimization framework is applied on real anatomical and implant data from 10 patients. The results demonstrate that, under clinically realistic circumstances, HDR-BT combined with simultaneous IHT using the TBT applicators has the potential to lead to a relevant therapeutic benefit in terms of OAR sparing or escalation of the equivalent physical dose.

## 2. Materials and Methods

### 2.1. Overview of Optimization Framework

**Figure 1:**
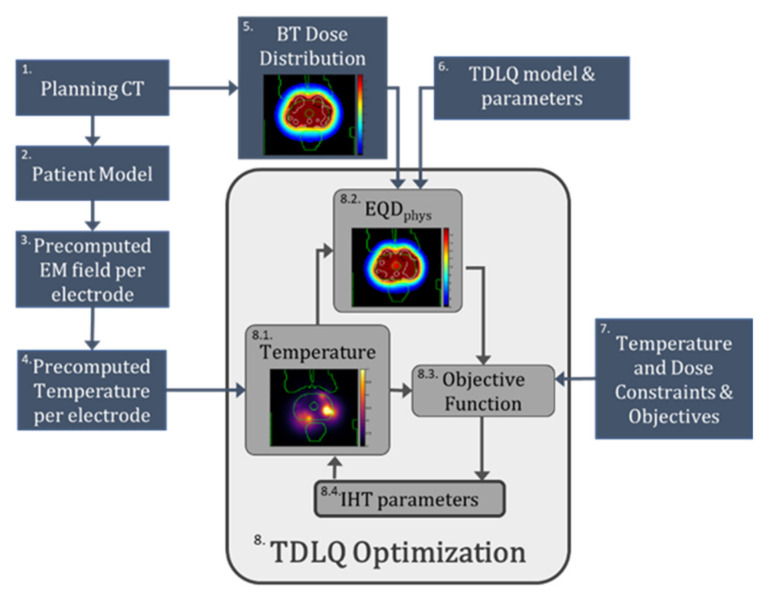
Graphical summary of the proposed optimization framework. 1. The planning CT is the initial input of the process. 2. The patient model is generated. 3. The EM field per electrode is precalculated. 4. The temperature distribution per electrode is precalculated. 5. The BT dose distribution is imported from the BT treatment planning software. 6. The TDLQ model is used for the calculation of the combined effect. 7. Both temperature and dose constraints and objectives are used for the optimization process. 8. The TDLQ optimization process optimizes the IHT parameters (8.4) that generate a temperature distribution (8.1) from which an EQD_phys_ distribution is generated (8.2). This EQD_phys_ distribution is used to compute the objective function, which needs to be minimized (8.3).

In the following paragraphs, we detail all steps that constitute the optimization framework (Figure 1). To reach the optimal thermoradiobiological TBT plan, first, the planning CT images (Figure 1 Item 1) are used to create the patient tissue model (Figure 1 Item 2). The position of the TBT applicators is reconstructed, and the electromagnetic (EM) fields and temperature distributions are precomputed per electrode (Figure 1 Items 3 and 4). The dwell times and dwell positions, defining the physical radiation dose, are optimized autonomously using the standard clinical HDR-BT protocol and workflow (Figure 1 Item 5). The IHT-TP parameters are then optimized based on the combined effect of the temperature distribution and the radiation distribution (Figure 1 Item 8). To evaluate the combined effect in terms of equivalent physical dose (EQD_phys_), the TDLQ model is applied (Figure 1 Item 6) with thermoradiobiological parameters from the literature. The optimization uses the standard clinical objectives and constraints as applied in the HDR-BT-only plans (Figure 1 Item 7).

### 2.2. Patient Anatomy Modeling

**Figure 2:**
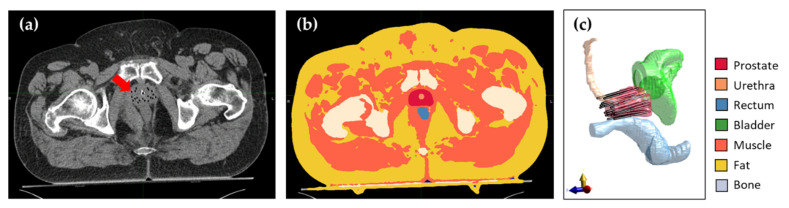
(**a**) Axial CT slice showing the anatomy of a patient with afterloading catheters (visible as black dots indicated by the arrow) inserted in the prostate. (**b**) Same axial slice of the resulting patient model after segmentation of all tissues. (**c**) Lateral 3D view of the prostate, OAR, and simulated TBT applicators in the same patient.

The patient tissues are modeled (Figure 1 Item 2) using information derived from computed tomography (CT) images taken as for HDR-BT planning (Figure 1 Item 1), with the dual function TBT applicators implanted into the patient. Prostate, urethra, rectum, bladder, bone, fat, muscle, and air volumes are distinguished. Prostate, urethra, rectum, and bladder volumes are defined by manual segmentation by an expert radiotherapy technologist using Oncentra^®^ Brachy (Elekta Brachytherapy Solutions, Veenendaal, The Netherlands), and approved by a radiation oncologist. For the other tissues, an automated workflow, based on thresholding, developed for clinical deep hyperthermia treatment planning is used (MIM Software, Cleveland, OH, USA). An example CT image with implanted afterloading catheters instead of TBT applicators can be seen in Figure 2a. The corresponding tissue model on the same slice can be seen in Figure 2b. The TBT applicator visualization and positioning are expected to be identical to the afterloading catheters used in standard HDR-BT treatment.

### 2.3. TBT Applicator Modeling, Positioning, and E-Field Calculation

Each TBT applicator consists of two 20-mm-long cylindrical electrodes with a 5-mm separation deposited on a needle-shaped flexible polyoxymethylene afterloading catheter and coated with a thin Parylene C coating. The two electrodes are connected to a power source through two feeding lines running parallel to the catheter up to the proximal end of the applicator. A detailed description of the applicator can be found in [25]. In the patient model, the TBT applicators are located and reconstructed as shown in the planning CT images (Figure 2c).

The electric field (E-field) is calculated for each electrode *i* as described in [35], using a finite element method solver for the electroquasistatic approximation [36]:

(1a) $$\nabla \cdot \;\left(\sigma +j2\pi f\varepsilon \right)\nabla V_{i}=0\;,$$

(1b) $$E_{i}=-\nabla V_{i}$$

where *σ* is the electrical conductivity, f is the frequency of the alternating E-field, *ε* is the dielectric permittivity, and *V_i_* is the scalar potential of electrode *i*. The E-field ***E_i_*** is calculated for each electrode from Equation (1b).

For a set of *n* different electrodes *i*, with amplitude settings $v_{i}$, all ***E_i_*** can be superposed to obtain the total E-field $E_{tot}$ as:

(2) $$E_{tot}=\overset{n}{\underset{i=1}{\sum }}E_{}\;v_{i}=Ev.$$

The power loss density (*P*) produced by the total field can then be derived from:

(3) $$P=\sigma \frac{{\left|E_{tot}\right|}^{2}}{2}=\frac{\sigma }{2}v^{H}E^{H}Ev=v^{H}Pv,$$

where **P** is an *n* × *n* matrix, and $E^{H}$ and $v^{H}$ are the Hermitian transpose of E and v, respectively.

For the E-field calculation (Figure 1 Item 3), the electric tissue properties are assigned according to the IT’IS database [37]. The electric properties of all used tissues, as well as those of the TBT applicator materials, are summarized in Table 1. All E-field and power calculations are performed in Sim4Life v6.2 (ZMT, Zurich, Switzerland).

### 2.4. Temperature Calculation and Superpositioning

The temperature distribution (*T*) resulting from all electrodes can be calculated by solving the Pennes’ bioheat equation [40,41]:

(4) $$\rho c\frac{\partial T}{\partial t}=\nabla \cdot \;\left(k\nabla T\right)+\rho Q+P-\rho_{b}c_{b}\rho \omega \left(T-T_{b}\right)$$

where *k* is the thermal conductivity, *c* is the specific heat capacity, *Q* denotes the specific metabolic heat generation rate, and *ω* is the perfusion rate. ρ is the mass density of the medium. $T_{b}$, $\rho_{b}$, and $c_{b}$ are the temperature, mass density, and specific heat capacity of blood, respectively. According to Das et al. [42], the temperature solution can be rewritten as:

(5) $$T=v^{H}Tv+T_{}$$

where **T**, similar to **P** in (3), is an *n* × *n* matrix, and *T_b_* is equal to the baseline temperature in the case of Dirichlet boundary conditions.

A fast optimization process is essential when applying simultaneous HDR-BT and IHT. To achieve faster temperature optimization, we use temperature superpositioning per electrode (Figure 1 Item 4), as proposed by Salgaonkar et al. for ultrasound-based IHT-TP [33]. In this method, all off-diagonal terms of **T** are neglected, reducing the complexity of the problem. With Δ*T_i_*, the temperature increase resulting from the power loss density *P_i_*, Equation (5) is simplified to:

(6) $$T=\overset{n}{\underset{i=1}{\sum }}v_{i}^{2}\Delta T_{}+T_{b}$$

Under the above assumption (temperature superpositioning per electrode), the temperature $T_{i}$ generated by each electrode *i* can be computed by solving the Pennes’ bioheat Equation as in (4). The precomputed temperature distributions per electrode were calculated using a finite element method (FEM) solver in Sim4Life v6.2 (ZMT, Zurich, Switzerland).

### 2.5. HDR-BT Treatment Plan and Dose Calculation

The HDR-BT treatment planning protocol is defined using dose–volume metrics. The prescribed radiation dose (*D_p_*) needs to be reached in at least 95% of the total target volume (*V*_100%_ ≥ 95%). For the OAR, the dose in a particular volume *x* (*D_x_*) is constrained to an organ-specific limit. The detailed HDR-BT treatment planning protocol is summarized in Table 2 and is based on [8].

The HDR-BT treatment plan (Figure 1 Item 5) is generated by expert radiotherapy technologists, based on inverse planning by simulated annealing [43] and manual finetuning, using the Oncentra^®^ Brachy treatment planning software, and reviewed by a radiation oncologist. The HDR-BT treatment plan is based solely on the radiation dose generated by an HDR ^192^Ir Flexisource, without considering the effect of the IHT. For the radiation dose calculation, a dose kernel based on the TG-43 standard is used [44].

### 2.6. Thermoradiobiological Modeling

To calculate the combined effect of the radiation and hyperthermia dose, thermoradiobiological modeling was performed (Figure 1 Item 6). The TDLQ model was applied [29]:

(7) $$S\left(D,T\right)=\exp\left[-\alpha \left(T\right)\;\cdot \;D-\beta \left(T\right)\;\cdot \;D^{2}\right],$$

where *S(D,T)* is the surviving fraction of tissue when simultaneously exposed to a radiation dose *D* and a temperature *T* for 1 h, assuming that the parameters *α(T)* and *β(T)* are exponentially dependent on the temperature according to:

(8a) $$\alpha \left(T\right)=\alpha \left(37\right)\cdot exp\left[\frac{T-37}{T_{ref}-37}\cdot ln\left[\frac{\alpha \left(T_{ref}\right)}{\alpha \left(37\right)}\right]\right],$$

(8b) $$\beta \left(T\right)=\beta \left(37\right)\cdot exp\left[\frac{T-37}{T_{ref}-37}\cdot ln\left[\frac{\beta \left(T_{ref}\right)}{\beta \left(37\right)}\right]\right],$$

where $T_{ref}$ is a reference temperature at which the $\alpha \left(T_{ref}\right)$ and $\beta \left(T_{ref}\right)$ have known values.

With this model, the equivalent dose received by a tissue, taking the thermal radiosensitization into account, is:

(9) $$EQD_{ref}=\frac{\alpha \left(T\right)\;\cdot \;D+G\cdot \beta \left(T\right)\;\cdot \;D^{2}}{\alpha \left(37\right)+\beta \left(37\right)\;\cdot \;d_{ref}}\;,$$

where *d_ref_* is the fraction dose to which the dose is normalized, and G is the Lea-Catcheside dose protraction factor, which is equal to 1 for a high dose rate source.

In our implementation, the equivalent dose is calculated normalized to the physical dose D. From Equations (8) and (9), this is calculated as:

(10) $$EQD_{phys}=\frac{\frac{\alpha \left(37\right)}{\beta \left(37\right)}exp\left[\frac{T-37}{T_{ref}-37}\cdot ln\left[\frac{\alpha \left(T_{ref}\right)}{\alpha \left(37\right)}\right]\right]\;\cdot \;D+exp\left[\frac{T-37}{T_{ref}-37}\cdot ln\left[\frac{\beta \left(T_{ref}\right)}{\beta \left(37\right)}\right]\right]\;\cdot \;D^{2}}{\frac{\alpha \left(37\right)}{\beta \left(37\right)}+D}\;$$

where the values of the radiobiological parameter *α*/*β* = *α(37)*/*β(37)*, and the thermoradiobiological parameters *α(T_ref_)*/*α(37)* and *β(T_ref_)*/*β(37)* for a given temperature *T_ref_* are needed for each tissue. The thermoradiobiological parameters for prostate tissue are assigned according to the in vitro data on the PC-3 and DU-145 prostate cancer cell lines in Pajonk et al. [16]. For normal tissues, there are no thermoradiobiological data available. Based on Overgaard [26], we can assume *α* and *β* parameters of normal tissue to have a similar thermal radiosensitization pattern as tumor tissue for the setting of simultaneous irradiation and hyperthermia; hence, we assigned the same *α(T_ref_)*/*α(37)* and *β(T_ref_)*/*β(37)* values to normal tissue. The radiobiological parameter *α*/*β* is conservatively set equal to 3 for all tissues at a normal temperature of 37 °C [45]. The values of all parameters are summarized in Table 3. Note that, in all following dose volume and dose coverage criteria in this article, dose is quantified in terms of *EQD_phys_*, which is temperature-dependent in the case of TBT and equal to the physical dose, D, in the case of BT only.

### 2.7. Thermoradiobiological Objective Function and Optimization Algorithm

For the optimization of the electrode amplitudes $v_{i}$ in the TBT treatment, an equivalent physical dose (*EQD_phys_*) based optimization algorithm is used (Figure 1 Item 8). The objectives are based on the criteria as reported in Table 2, combined with an overall upper temperature limit of *T_max_* = 47.5 °C [20]. This is formulated in a minimization problem containing penalty functions *PF_i_* for every violated constraint *i*, and objective scoring functions *SF_j_* that return lower values for better performance for an objective *j*:

(11) $$\Omega =W\underset{i}{\sum }PF_{i}+\underset{j}{\sum }SF_{j}\;.$$

The penalty functions *PF_i_* are in the form of:

(12a) $$PF_{i}=\max\left[0,p_{i}\left(C_{}-L_{i}\right)\right],\;$$

where the values for *C_i_* and *L_i_* are according to Table 4 and the polarity factor *p_i_* is +1 for low pass constraints and −1 for high pass constraints. The scoring functions *SF_j_* are in the form of:

(12b) $$SF_{i}=-w_{j}\left|O_{j}-G_{j}\right|\;,$$

where the values for *O_i_*, *G_j_*, and *w_j_* are according to Table 4 (Figure 1 Item 7). The penalty weight factor *W* is set to a constant *W* = 10^3^ for all constraints in order to ensure a high penalty for every constraint violation. The objective function *Ω* is minimized using a particle swarm optimization algorithm in MATLAB (MathWorks Inc., Natick, MA, USA).

### 2.8. Temperature Superpositioning Validation

While the temperature superpositioning method for fast temperature calculations has been validated for interstitial ultrasound power sources [33], we also perform a validation in our approach. To validate the accuracy of the temperature superpositioning method, we calculated a temperature distribution using the superpositioning method and we recalculated the temperature distribution resulting from the same power amplitudes using the FEM solver. In this way, we investigated the assumption of Equation (4) that the off-diagonal terms in **T** not contributing significantly to the total temperature distribution is correct. The agreement between the two calculation methods was scored using three-dimensional γ-index analysis [46,47].

### 2.9. Implementation on Patient Data

To evaluate the proposed TBT treatment plan, we used data of 10 prostate cancer patients treated at our institution with HDR-BT monotherapy in two fractions of *D_p_* = 13.5 Gy in a single day with a single implantation. For the first fraction, the patients were treated with a US-based HDR-BT treatment and, for the second fraction, they were treated with a CT-based HDR-BT treatment [35]. To validate the implementation of our treatment plan, we simulated a replacement for the second fraction by a TBT treatment and compared the resulting *EQD_phys_* distribution to the original HDR-BT-only physical dose distribution by assuming that the TBT applicators are placed at the same position as the flexible 6F ProGuide afterloading needles (Elekta Brachytherapy Solutions, Veenendaal, The Netherlands).

**Figure 3:**
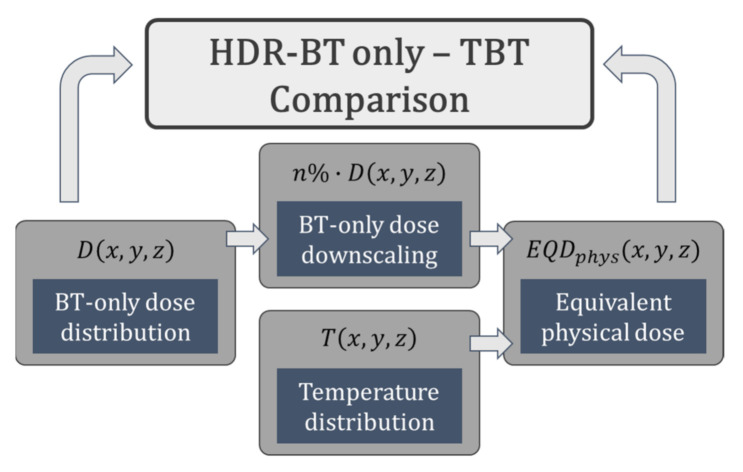
Flowchart illustrating how the TBT *EQD_phys_* distribution is generated from and compared with the original BT-only dose distribution.

The TBT treatment plan used a uniformly scaled-down version of the original HDR-BT dose distribution that had been clinically generated [35]. Different plans were generated using various combinations for the thermoradiobiological parameters *α(43)*/*α(37)* and *β(43)*/*β(37)*, according to Table 3. The BT dose distributions were scaled from 70% to 95% of the original clinical dose. This process is illustrated in Figure 3.

## 3. Results

### 3.1. Temperature Superpositioning Validation

**Figure 4:**
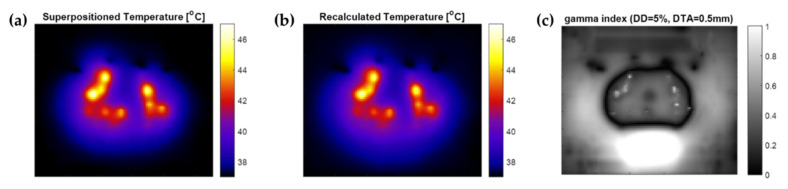
Comparison between superpositioned temperature calculation and FEM recalculation on the central axial slice in the prostate. (**a**) Temperature map using superpositioning of separate FEM calculations for each electrode. (**b**) Temperature map using a single FEM calculation for the same, combined, electrode settings. (**c**) γ-index map of the comparison.

The temperature distribution in a simulated patient with 18 applicators with given electrode amplitudes was calculated using both the superpositioning method (Figure 4a) and an FEM recalculation (Figure 4b). With the FEM-recalculated temperature as a reference, a γ-index analysis was performed. Applying 5%/0.5 mm dose difference and distance to agreement criteria, a passing rate >95% was observed, suggesting a good match to the reference (Figure 4c). As can be seen in Figure 4c, the highest gamma index values were positioned in the far-field of the temperature increase, where temperature values were low.

### 3.2. Thermal Radiosensitization

**Figure 5:**
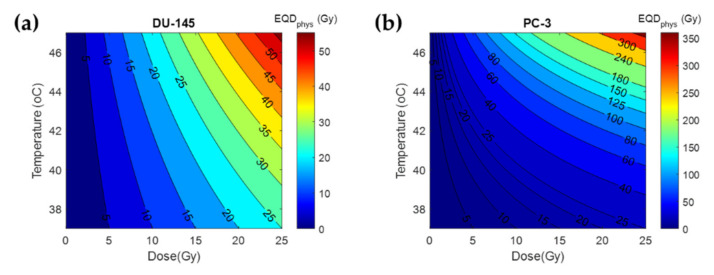
Isodose curves for the *EQD_phys_* resulting from different combinations of physical dose and temperature for 1 h: (**a**) *EQD_phys_* assuming DU-145 data; (**b**) *EQD_phys_* assuming PC-3 data.

To illustrate the thermal radiosensitization that is expected in prostate cancer cells, we applied the values of Table 3 to Equation (10) and visualized the results for different radiation doses and temperatures. How those values affect the *EQD_phys_* dose resulting from a TBT treatment can be seen in Figure 5. As can be seen, there is a considerable difference in thermal radiosensitization between the PC-3 and DU-145 cell lines. Indicatively, the thermal enhancement caused by a 43 °C temperature in PC-3 is approximately threefold that of DU-145.

### 3.3. Treatment Planning Results

**Figure 6:**
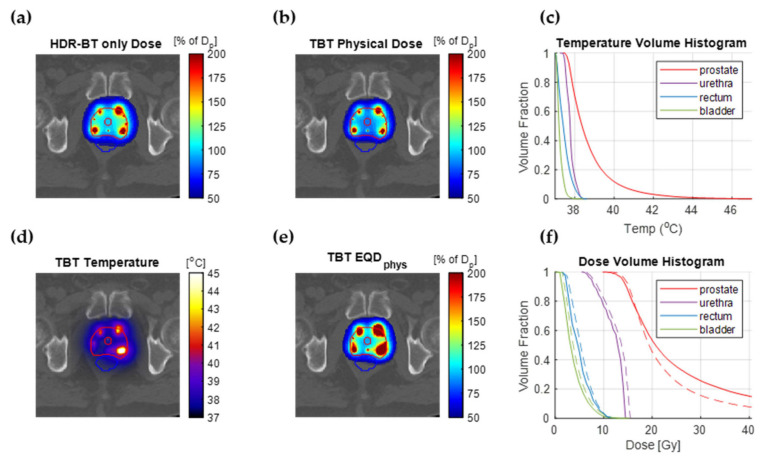
TBT TP results assuming thermoradiobiological parameters equal to the average between DU-145 and PC-3 data. (**a**) The original clinically applied HDR-BT dose fraction. (**b**) The applied TBT physical dose (80% of original). (**c**) Temperature volume histogram showing the temperature coverage in the prostate and OARs. (**d**) The temperature distribution calculated for the optimal TBT plan. (**e**) The TBT *EQD_phys_* resulting from the combined treatment. (**f**) Dose volume histogram of the prostate and OARs for the HDR-BT-only dose (dashed line) and the TBT *EQD_phys_* dose (solid line).

We optimized the temperature distribution for the 10 simulated patient plans for different scalings of the HDR-BT dose. Figure 6 shows the results for a single patient with an HDR-BT dose scaling of 80%. The *α(43)*/*α(37)* and *β(43)*/*β(37)* values are assumed equal to the average between DU-145 and PC-3 data, which gives *α(43)*/*α(37)* = 1.6 and *β(43)*/*β(37)* = 4.3. The *EQD_phys_* volume histogram shows that the same target coverage is reached (96.6%), while D_0.1cc_ for the urethra, and *D*_1*cc*_ for the rectum and bladder are reduced by 6.1%, 4.9%, and 8.2% of the prescribed dose, respectively. On the other hand, the *V*_150*%*_ and *V*_200%_ are higher by 12.1% and 12.4% of prostate volume, respectively.

**Figure 7:**
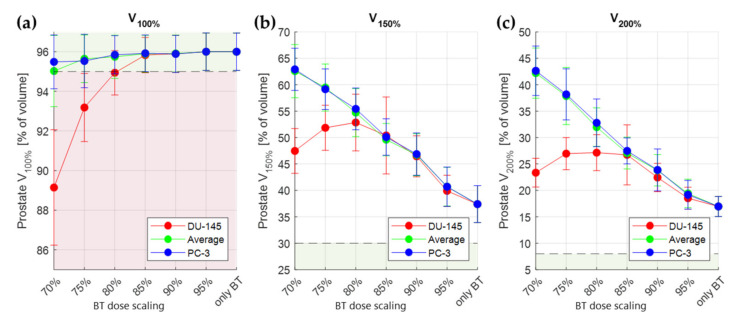
Average values (±standard deviation) over 10 simulated patients of TBT prostate *V*_100%_ (**a**), *V*_150*%*_ (**b**), and *V*_200%_ (**c**) for different scalings of the original HDR-BT dose. The different colors correspond to the plans generated based on different thermoradiotherapeutic values (red for DU-145, blue for PC-3, and green for the average between DU-145 and PC-3). It is evident that the original prostate coverage is met when the physical dose is scaled over 80% of the original value. The vertical bars correspond to standard deviation. The horizontal dashed lines correspond to the objective (*V*_100%_) and soft constraint (*V*_150*%*_ and *V*_200%_) limits. The green and red areas correspond to targeted and constrained values, respectively.

Figure 7 summarizes the results over all simulated patients (showing average values and standard deviations) for the prostate. From this figure, it can be seen that the required target coverage can be reached when using at least 80% of the original physical dose of the HDR-BT-only treatment (Figure 7a) for the less thermosensitive DU-145 cells. This result is valid for all three evaluated values of *α(43)*/*α(37)* and *β(43)*/*β(37)*: PC-3, DU-145, and average. For radiosensitization according to the more thermosensitive PC-3 cells, this is as low as 70% of the original physical dose. It is evident that the addition of IHT contributes to considerably higher values for *V*_150*%*_ and *V*_200%_. This should be expected, since the IHT sources and HDR-BT sources irradiate from the same positions: the TBT applicator.

**Figure 8:**
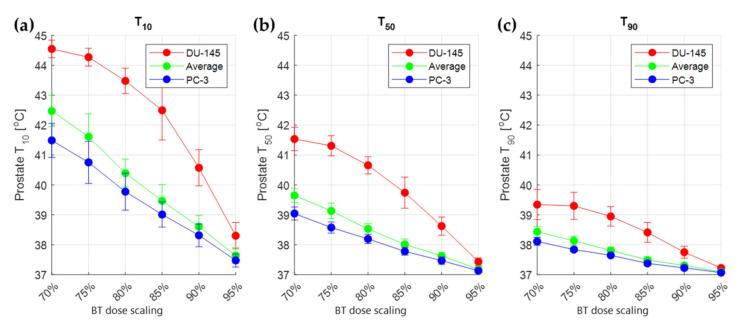
Average values (±standard deviation) over 10 simulated patients of the optimal TBT prostate *T*_10_ (**a**), *T*_50_ (**b**), and *T*_90_ (**c**) for different scalings of the original HDR-BT dose. The different colors correspond to the plans generated based on different thermoradiotherapeutic values (red for DU-145, blue for PC-3, and green for the average between DU-145 and PC-3). The vertical bars correspond to standard deviation.

Figure 8 shows the *T*_10_, *T*_50_, and *T*_90_ values (temperature reached in at least 10%, 50%, and 90% of the total volume, respectively) over all simulated patients. It is evident that higher *T*_10_, *T*_50_, and *T*_90_ values are required for lower physical doses and for lower thermal sensitivity of the tumor. Furthermore, *T*_50_ and *T*_90_ values are mainly under the 39 °C value. This means that hyperthermia values are not needed in the whole prostate to reach the optimal *EQD_phys_* distribution. On the other hand, the temperature is, as is the radiation dose, per definition, heterogeneous in the prostate.

**Figure 9:**
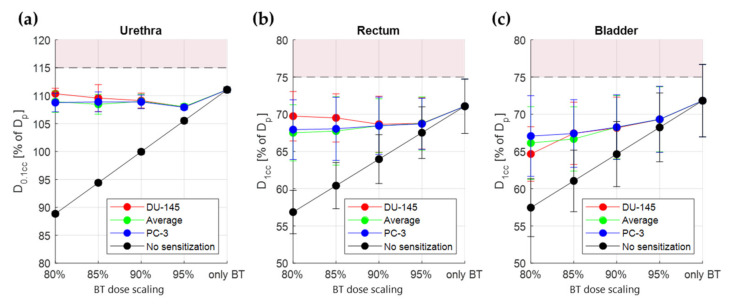
Average values (±standard deviation) over 10 simulated patients of the TBT TP parameters for different scaling of the original dose: urethra *D*_0.1*cc*_ (**a**), rectum *D*_1*cc*_ (**b**), and bladder *D*_1*cc*_ (**c**). The different colors correspond to the plans generated based on different thermoradiotherapeutic values (red for DU-145, blue for PC-3, and green for the average between DU-145 and PC-3). The black line shows the lowest possible value, assuming no radiosensitization of the normal tissue. The horizontal dashed lines correspond to the constraint limit for each criterion. The red areas correspond to constrained values for each criterion.

For the OAR there are no data available on their sensitivity. Therefore, we evaluated the dose metrics for two extreme cases: assuming sensitization as high as in tumor tissue [26] and assuming no sensitization. The actual sensitization is expected to be somewhere in between the two extreme values. The OAR dose metrics reached with the different TBT plans are visualized in Figure 9. For all evaluated cases, the *D*_0.1*cc*_ of the urethra, the *D*_1*cc*_ of the rectum, and the *D*_1*cc*_ of the bladder are lower with the TBT plan than with the HDR-BT-only plan (*p*-value < 0.001, paired two-sided Wilcoxon signed rank test).

## 4. Discussion

Extensive biological studies have indicated that hyperthermia is a potent sensitizer to radiotherapy, especially when applied simultaneously with the radiation dose [12,13]. To benefit from the high radiosensitization achieved in such a setting, both the thermal and radiation dose have to be focused sufficiently well to the target. In the TBT setting, both doses are administered from within the target region, which makes it the ideal method for simultaneous application. The highly localized deposition of both doses requires, however, good planning of the electrode amplitudes, dwell times, and dwell positions for good thermal and radiation coverage of the target and OAR sparing.

From a thermoradiobiological point of view, three-dimensional evaluation of combined radiotherapy and hyperthermia treatments is possible using the TDLQ model [29]. It is challenging to meet the set dose targets and constraints with the resulting *EQD_phys_* without optimizing the temperature distribution according to those criteria. Given the high number of variables that need to be tuned, an automated method to optimize the temperature distribution is necessary for an optimal TBT treatment. Therefore, with the proposed optimization method, we can optimize the temperature on radiotherapeutic dose criteria.

To produce fast calculations of the temperature distribution, temperature superpositioning was used. We showed in our evaluation that this is a reasonable estimation of the temperature distribution, with a passing rate >95% for 5%/0.5 mm dose difference and distance to agreement criteria. It is important to note that the temperature calculation method showed the best results where high temperature elevations and consequently high radiosensitization were present (Figure 4), with the lower temperature regions mainly in the rectum showing less agreement with the single FEM temperature calculation. Therefore, with the current temperature calculation method, simulated rectum temperatures might not be reliable enough. For a more accurate estimation of the final temperature distribution, one could consider recalculating the final temperature distribution based on the optimal IHT-TP settings for evaluation purposes. Another option is to apply the method of Das et al. [42] (Equation (4)), although it would lead to slower optimizations due to the high number of electrodes producing an E-field.

We showed in our results (Figure 7) that the calculated TBT *EQD_phys_* distribution can meet the prostate coverage *V*_100%_ ≥ 95% for different values of *α(43)*/*α(37)* and *β(43)*/*β(37)* with up to 20% decrease in physical dose (80% of original HDR-BT dose). For the required temperature elevations (Figure 8), it is evident that temperature homogeneity in the target (Figure 6d) is not necessary to meet the prescribed target coverage. In the clinical feasibility study of the MECS applicator by van Vulpen et al. [48], it was noted that the observed high temperature gradients (*T*_10_ = 45.7 °C, *T*_90_ = 39.4 °C) were an undesired effect. By looking at the temperature distribution as a radiation dose sensitizer, we see that we can still reach significant improvements to the treatment, since only underdosed regions of the target are in need of a temperature increase.

Since there is no information available about the radiosensitization of normal tissues, we assumed in our optimization process a worst-case scenario of equal radiosensitization for normal and tumor tissue. With this assumption, we saw *EQD_phys_* sparing for all three OAR (2.2 ± 1.7%, 2.6 ± 2.1%, and 4.2 ± 2.2% decrease for urethra *D*_0.1*cc*_, rectum *D*_1*cc*,_ and bladder D_1cc_, respectively). In practice, we can expect a lower normal tissue radiosensitization than tumor tissue radiosensitization, especially since human prostate cancer cells are remarkably thermosensitive [15]. Therefore, we also evaluated the maximum potential decrease in the OAR by assuming no thermal radiosensitization in normal tissue (Figure 9). It is evident that OAR sparing can be significant in such a scenario (i.e., urethra *D*_0.1*cc*_ as low as 90% of *D_p_*). To draw definitive conclusions on the level of OAR sparing, the availability of thermoradiobiological data for normal tissues is an absolute requirement. Should the OAR sparing be insufficient, one can also investigate OAR cooling. Another option would be to investigate whether sequential TBT is beneficial. However, in sequential TBT the level of tumor radiosensitization is lower and the sequential procedure could prolong overall treatment time.

While OAR sparing can be expected, it is also evident from Figure 7 that the high target doses (*V*_150*%*_ and *V*_200%_) become even higher in the TBT setting. Namely, scaling down the physical dose increases the *V*_150*%*_ and *V*_200%_ to a saturation point where the *V*_100%_ target can be reached (for DU-145, this is visible at 80% target coverage in Figure 7). This effect is expected, given the fact that both modalities are delivered to the target from within the same applicator. Whether this is a negative effect or not can be debated. On the one hand, clinical treatment protocols strive to decrease extreme heterogeneity in the tumor by applying soft constraints on the high prostate doses, as is also carried out in the current study [6,49]. On the other hand, guidelines on prostate HDR-BT do not restrict high doses [10,11]. This can be explained by the fact that a saturation dose beyond which no further injury can occur likely exists in prostate brachytherapy [50,51].

In this study, we only optimize the temperature distribution based on the combined treatment. We expect that also optimizing the radiation dose distribution based on the combined effect has the potential to lead to more enhancement, i.e., better results, given the higher number of degrees of freedom. This additional optimization opportunity should, therefore, be considered in future research.

The TDLQ model is not complete in describing the combined effect of radiation and temperature elevation. The extended TDLQ model incorporating direct temperature-induced cytotoxicity has also been proposed and evaluated for cervical cancer cell lines [30]. There are currently not enough data to apply the same model for prostate cancer. However, in our application, where very high radiation fraction doses are applied, it can be presumed that most of the cell death is caused by radiation rather than temperature increase. In any case, should there be enough radiobiological data available, a more elaborate model could easily be applied to this algorithm as well.

In Figure 8, we showed that the calculated *T*_10_, *T*_50_, and *T*_90_ values needed for sufficient target coverage are, in some cases, lower than what is commonly regarded as adequate temperature elevation in hyperthermia treatments. These values are, however, set for an IHT treatment duration of 1 h. It is debatable whether temperatures under 39 °C can cause tumor radiosensitization at all [52]. One can, therefore, choose to normalize the length of the treatment to achieve the same thermal dose in, i.e., *CEM43* [12] or *AUC* [52]. This is also convenient in a simultaneous TBT treatment, since an HDR-BT treatment has a delivery time of approximately 10–20 min.

We have presented a method for automated IHT-TP optimization based on thermoradiotherapeutic criteria when IHT is used simultaneously with HDR-BT. We also showed that the results of the optimization are very dependent on the thermosensitivity of the tumor and normal tissue. With information on thermosensitivity of the involved tissues not yet available, the full potential use of this algorithm still needs to be determined. However, it can already serve as a promising tool for further development of IHT in combination with HDR-BT.

## 5. Conclusions

In this study, we presented a framework to optimize the temperature for simultaneous HDR-BT and IHT, based on the resulting *EQD_phys_* of the combined treatment. This gives the opportunity of treatment planning on the same radiotherapeutic dose constraints and objectives as for HDR-BT only. We established that the fast calculation of the temperature distribution is accurate. Furthermore, on a sample of 10 patients, the calculated equivalent dose distribution predicts a favorable reduction in the dose in OARs. At the same time, the target dose coverage remains at the same level as prescribed in the standard protocol, while the high-dose regions (*V*_150*%*_ and *V*_200%_) get considerably higher values. While this framework offers a valuable tool for simultaneous thermobrachytherapy treatment plan optimization, further research on the biological effects of both heat and radiation is needed to confirm the clinical relevance of a simultaneous thermobrachytherapy treatment.

## Figures and Tables

**Table 1:** Electric and thermal properties of the applicator materials and the tissues used in the simulations.

Tissue	*ρ* (kg/m^3^)	*σ* @27 MHz (S/m)	ε_r_ @27 MHz	*c* (J/kg/K)	*k* (W/m/K)	ω (ml/kg/min)
Applicator Dielectric [38,39]	1289	1 × 10^−5^	2.4	712	0.084	-
Air [37]	1.164	0	1.0	1004	0.0273	-
Muscle [37]	1090.4	0.654	95.8	3421	0.495	40
Fat [37]	911	0.061	17.9	2348	0.211	33
Bone [37]	1908	0.052	21.8	1313	0.320	10
Prostate [37]	1045	0.838	120.1	3760	0.512	394
Rectum [37]	1045	0.654	95.8	3801	0.557	0
Urethra [37]	1102	0.375	88.8	3306	0.462	394
Bladder [37]	1086	0.276	31.5	3581	0.522	78

**Table 2:** Fraction dose objectives and constraints of the clinical HDR-BT protocol.

Tissue	Criterion	Aim	Type
Prostate	*V*_100%_	≥95%	Objective
	*V*_150*%*_	<30%	Soft Constraint
	*V*_200%_	<8%	Soft Constraint
Urethra	*D*_0.1*cc*_	<115%	Hard Constraint
Rectum	*D*_1*cc*_	<75%	Hard Constraint
Bladder	*D*_1*cc*_	<75%	Soft Constraint

**Table 3:** Thermoradiobiological parameters applied in this study, with *T_ref_* = 43 °C.

Tissue	*α(37)*/*β(37)*	*α(43)*/*α(37)*	*β(43)*/*β(37)*
**Prostate**	3		
PC-3 [16]		2.4	6.8
DU-145 [16]		0.8	1.8
**Normal Tissue**	3		

**Table 4:** Constraints and objectives applied in the objective function.

Constraints	Tissue	Criterion (*C_i_*)	Limit (*L_i_*)	Type
	Prostate	V_100%_	Value of BT-only	High pass
	Urethra	D_0.1cc_	Value of BT-only	Low pass
	Rectum	D_1cc_	Value of BT-only	Low pass
	Bladder	D_1cc_	Value of BT-only	Low pass
	All	T_max_	47.5 °C	Low pass
**Objectives**	**Tissue**	**Criterion (*O_j_*)**	**Goal (*G_j_*)**	**Weight (*w_i_*)**
	Urethra	D_0.1cc_	0	1
	Rectum	D_1cc_	0	1
	Prostate	V_150%_	30% of volume	0.01

## Data Availability

The data presented in this study can be made available upon request to the corresponding author.
